# Middle Cerebral Artery Duplication: A Near Miss for Stroke Thrombectomy

**DOI:** 10.7759/cureus.15220

**Published:** 2021-05-24

**Authors:** Elliot Pressman, Sheyar Amin, Swetha Renati, Maxim Mokin

**Affiliations:** 1 Neurosurgery & Brain Repair, University of South Florida Health, Tampa, USA; 2 Neurology, University of South Florida Health, Tampa, USA

**Keywords:** anatomic variant, duplication, middle cerebral artery, stroke, thrombectomy

## Abstract

Middle cerebral artery (MCA) duplication is a rare anatomical arrangement where an anomalous MCA arises from the distal end of the internal carotid artery. If occluded, a duplicated MCA can present with significant deficits comparable to an occlusion of the M2 vessel without obvious findings on vessel imaging via computed tomography angiography (CTA) or magnetic resonance angiography.

A female in her 30s with no past medical history presented with suspected acute stroke 8 hours after last known normal-featuring new-onset right-sided weakness, facial droop, and slurred speech, which corresponds to a National Institutes of Health Stroke Scale score of 13. Head CTA was interpreted as preserved patency of intracranial vessels. CT perfusion was suggestive of a large area of penumbra. Emergent cerebral angiography demonstrated MCA duplication on the left side with proximal occlusion of the M1 branch supplying the traditional M2 territory. Mechanical thrombectomy achieved grade TICI 2b reperfusion. Throughout her hospital stay, her aphasia started to resolve, and the patient was discharged to inpatient rehabilitation.

This case presents a diagnostic challenge and learning point in identifying similar patients in the future. We suggest the clinician, given a high clinical suspicion for large vessel occlusion, even if CTA is negative, to continue with CT perfusion to not miss stroke in patients with MCA duplication. If CT perfusion shows substantial deficit in an MCA distribution, one must consider that the patient may have an MCA duplication.

## Introduction

In 1973, Teal et al. defined a middle cerebral artery (MCA) duplication as an anatomical arrangement where an anomalous MCA arises from the distal end of the internal carotid artery (ICA) [1]. The frequency of this anatomic variant is reported to be between 0.2% and 2.9% and has only been reported in the literature in regard to being found during work-up for associated aneurysms [2,3]. When acutely obstructed, a duplicated MCA can present with significant deficits, such as hemiparesis and aphasia akin to an ‘M2-like’ syndrome [4]. However, despite the potential for significant deficits, during work-up of a typical stroke, vessel imaging via computed tomography angiography (CTA) or magnetic resonance angiography may miss the obstruction given the patency of the second MCA. No previous cases have been reported with an obstruction of this kind.

This discrepancy between the clinical presentation and imaging findings can lead to a delay in revascularization, and in turn potentially reversible deficits becoming permanent. In the report below, we describe a case with an acutely obstructed, duplicated MCA in a patient who then received mechanical thrombectomy to bring awareness of this possibility to readers to avoid missing similar arrangements.

## Case presentation

**Figure 1:**
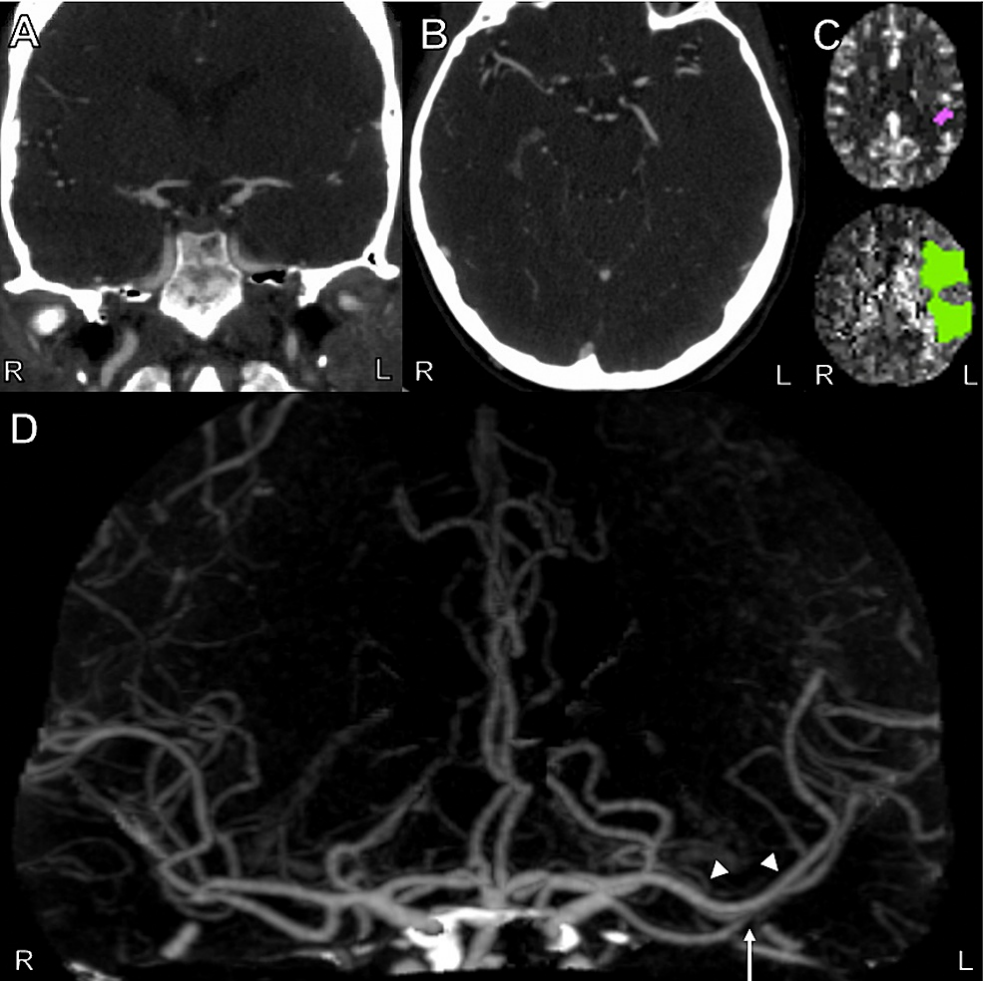
CTA and CT Perfusion Images of the Head from the Patient's Initial Work-up A: Coronal view of CTA scan of the head showing what appears to be normal flow through the right and left middle cerebral artery M1 segments. B: Axial view of CTA scan of the head showing what appears to be normal flow through the right and left middle cerebral artery M1 segments. C: Axial view of CT perfusion scan of the head showing core infarct (purple) and ischemic penumbra (green). D: Anteroposterior view of three-dimensional reconstruction of CTA of the head showing occluded left middle cerebral artery M1 segment (white arrow). The arrowheads point to a second MCA branch. Note the 'normal' appearance of MCA on the opposite (right) side. CTA, computed tomography angiography; MCA, middle cerebral artery.

A female in her 30s with no reported past medical history presented to the emergency department (ED) with suspected acute stroke 8 hours after her last known normal. She had awoken with new-onset right-sided weakness, facial droop, and slurred speech, corresponding to a National Institutes of Health Stroke Scale (NIHSS) score of 13. While in the ED, she became increasingly confused with new-onset aphasia resulting in further worsening of her NIHSS. Emergent non-contrast head CT showed a small area of hypodensity in the left parietal lobe. Head CTA was obtained and was interpreted by an on-call radiologist as preserved patency of intracranial vessels though later re-interpretation with hindsight revealed a proximal MCA occlusion as seen in Figure 1. CT perfusion showed a large area with increased mean transit time with a small amount of decreased cerebral blood volume in the left cerebral hemisphere with a mismatch ratio of 17 suggesting a large area of penumbra, as shown in Figure 1C. Though the CTA did not demonstrate a significant proximal occlusion of the MCA, given the extensiveness of this patient’s deficits, and the large left hemispheric penumbra on CT perfusion, the patient was taken for emergent cerebral angiography with possible stroke thrombectomy.

**Figure 2:**
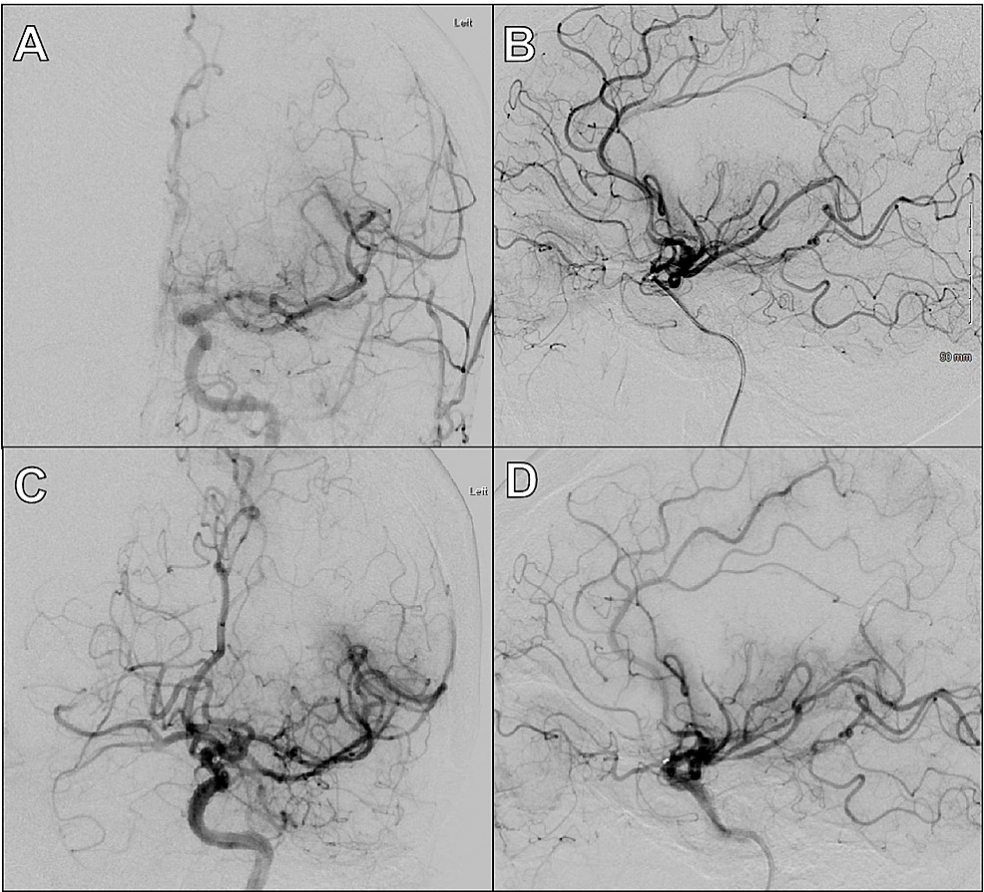
Representative Images from Patient's Cerebral Angiography and Thrombectomy A: Pre-thrombectomy anteroposterior view of digital subtraction angiography of the head during contrast injection in the left ICA showing occluded duplicated M1. B: Pre-thrombectomy lateral view of digital subtraction angiography of the head during contrast injection in the left ICA showing occluded duplicated M1. C: Post-thrombectomy anteroposterior view of digital subtraction angiography of the head during contrast injection in the left ICA showing TICI 2b recanalization because of distal emboli and incomplete thrombectomy. D: Post-thrombectomy lateral view of digital subtraction angiography of the head during contrast injection in the left ICA showing TICI 2b recanalization because of distal emboli and incomplete thrombectomy. ICA, internal carotid artery.

Cerebral angiography demonstrated that the patient had MCA duplication on the left side, with proximal occlusion of one of the two M1 branches as seen in Figure 1D and Figure 2A,B, explaining the patient’s symptoms and perfusion deficit seen on CT perfusion. The occluded M1 trunk appeared to supply the territory typically supplied by the superior M2 branch. With mechanical thrombectomy, grade TICI 2b reperfusion was achieved (as seen in Figure 2). Throughout her hospital stay, her aphasia started to resolve, and the patient was discharged to inpatient rehabilitation without complications.

## Discussion

This case demonstrates the difficulties in diagnosing a proximal MCA stroke in a patient with MCA duplication. Prompt diagnosis of this emergent large vessel occlusion (ELVO) is vital so that the patient can be taken for mechanical thrombectomy as soon as possible to restore perfusion to the penumbra before temporary deficits become permanent. 

In the above-mentioned patient, though there was high clinical suspicion for an ELVO given her hemiparesis and aphasia, CTA was initially interpreted as no occlusion since the second MCA on that side was still patent. However, the CT perfusion scan revealed a large area of ischemic penumbra, suspected at the time to be due to an ELVO not shown on the CTA. The diagnosis of proximal MCA occlusion in one of two left MCAs was then made by digital subtraction angiogram (DSA). Only after a thorough review of the initial CTA under the bias of hindsight were we able to identify the second MCA with proximal occlusion superimposed with the ‘normal’-appearing MCA as demonstrated in Figure 1. Of note, the initial CTA/CT perfusion (with RAPID) was reviewed by the stroke team, radiologist, and the interventionalist. 

The case described above presents a diagnostic challenge in identifying similar patients in the future. Since the duplicated MCA arises from the ICA terminus, it is easy for a practitioner to glance at the CTA and note patency of the intracranial vessels without catching the obstructed anomaly. To prevent this, we suggest the clinician, who has a high clinical suspicion for ELVO, as in the case described above, even if a CTA is negative, to continue with CT perfusion so as to not miss a stroke in a patient with MCA duplication or another similar anomaly. Automated applications for ELVO detection such as those provided by RAPID.AI and VIZ.AI are designed to automatically identify potential thrombectomy-eligible cases on CT or MR angiogram images [5,6]. While artificial intelligence-driven algorithms may improve ELVO detection, physicians managing acute stroke cases should be mindful that cases with rare anatomical variants such as the one reported here may not be included in training or validation protocols, increasing the possibility of false-negative findings [7].

Though this report details merely one patient’s presentation, it suggests that if CT perfusion shows substantial deficit in an MCA distribution, one must consider that the patient may have an MCA duplication, or other rare anatomic variant, thus skewing the normal interpretation of the CTA. They should then be taken for emergent DSA and thrombectomy to preserve the susceptible penumbra seen on CT perfusion.

## Conclusions

MCA duplication is a rare anatomical arrangement where an anomalous MCA arises from the distal end of the ICA. Timely identification of an acutely occluded duplicated MCA is critical in reversing its ‘M2-like’ neurologic deficits. Recognition of this anatomic variant is challenging because in these patients CTA appears to show patency of all intracranial vessels.
